# Image Registration Algorithm Using Mexican Hat Function-Based Operator and Grouped Feature Matching Strategy

**DOI:** 10.1371/journal.pone.0095576

**Published:** 2014-04-21

**Authors:** Feng Jin, Dazheng Feng

**Affiliations:** 1 School of Computer Science and Technology, Xidian University, Xi’an, China; 2 National Key Laboratory of Science and Technology on Radar Signal Processing, Xidian University, Xi’an, China; Universitat Rovira i Virgili, Spain

## Abstract

Feature detection and matching are crucial for robust and reliable image registration. Although many methods have been developed, they commonly focus on only one class of image features. The methods that combine two or more classes of features are still novel and significant. In this work, methods for feature detection and matching are proposed. A Mexican hat function-based operator is used for image feature detection, including the local area detection and the feature point detection. For the local area detection, we use the Mexican hat operator for image filtering, and then the zero-crossing points are extracted and merged into the area borders. For the feature point detection, the Mexican hat operator is performed in scale space to get the key points. After the feature detection, an image registration is achieved by using the two classes of image features. The feature points are grouped according to a standardized region that contains correspondence to the local area, precise registration is achieved eventually by the grouped points. An image transformation matrix is estimated by the feature points in a region and then the best one is chosen through competition of a set of the transformation matrices. This strategy has been named the Grouped Sample Consensus (GCS). The GCS has also ability for removing the outliers effectively. The experimental results show that the proposed algorithm has high registration accuracy and small computational volume.

## Introduction

### A. Background

Image registration is the process of matching and fusion of multiple images taken from the same scene, at different times, by different sensors, and from different perspectives [1]. It is a hot spot on the computer vision, pattern recognition, medical image processing and remote sensing data processing. Image registration is widely used in the multi-source remote sensing data integration and analysis, motion tracking of small target under complex scenes, matching of landscape and map, image stitching and topographic height reconstruction. Currently, in a wide range of applications of image registration, ones often adopt methods based on the image feature extraction.

According to different classes of image features, the methods can be divided into the area-based and the point-based. A classical local area-based method is a combination of chain code and invariant moment proposed by Dai and Khorram [2]. The improved Laplacian of Gaussian (LoG) operator is used for the extraction of the area contours, and the contours are further described by the chain code.

The feature points are also called the interesting points or key-points. The feature point-based methods are widely used, such as the scale invariant feature transform (SIFT) operator, proposed by Lowe [3], and the Harris-Laplace operator which is the improved Harris operator with scale invariance proposed by Mikolajczyk and Schmid [4]. These two operators defined in the scale space are the most classical application of the Gaussian kernel filter.

These two classes of methods, however, both have inherent shortcomings that need to be dealt with. The performances of the local area-based methods are highly influenced by the accuracy of the LoG operator, and they behave when the shape of objects is seriously changed in the matching images. For example, the fields or lakes frequently change their area along with the time lapses. The even worse is that it cannot provide sufficient features to support the registration of images with complex texture or perform the 3-D object reconstruction. On the other hand, the point-based methods have higher accuracy, their ability for differentiating and localizing the points depend on the complex description of the point properties. For example, the SIFT operator describes each feature point with a 128-D vector.

### B. Literature Review

The SIFT and Harris-Laplace operator are the most classical methods of scale invariant points detection and matching. They are based on the theory of scale-space analysis. There are many other algorithms developed in the theory and techniques, such as the speedup robust features [5] (SURF) and the PCA-SIFT [6]. Recently, Padmavathi, Muthukumar and Thakur [7] proposed a method by combining the Kernel PCA (KPCA) and SIFT together (called KPCA- SIFT feature detection) for underwater images. The method focuses on the approaches to the KPCA using reproduced kernels. Hence, KPCA is used for feature extraction and dimension reduction of SIFT. Cui and Ngan [8] developed multiple fan sub regions named Fan features depict the image neighborhood of a key point. The Fan features are made scale-invariant by using the automatic scale selection method based on Fan Laplacian of Gaussian (FLOG).

Instead of feature point-based approach, Tuytlelaars and Gool [9] defined and extracted an intensity-based, local affine invariant region that is independent of the presence of edges or corners in the image. Such regions are also applied in wide baseline stereo matching. Reference [10] proposed an efficient and practically fast detection algorithm for detecting the maximally stable extremal regions (MSER). And then the invariants from multiple measurement regions are used to establish tentative correspondences. Kadir, Brady and Zisserman [11] developed a novel algorithm called Scale Saliency for quantifying image region saliency. In their approach, regions are considered salient if they are simultaneously unpredictable both in some feature and scale-space.

There is also a class of image registration algorithms based on the spatial relations or constraints among points, which is receiving much more attention. These methods are widely used in image classification, pattern recognition and object recognition. Reference [12] provides a matching method which is to find the correspondence between groups of contour points. Two groups are considered to be matched when the two point sequences formed by the two groups lead to a perfect one-to-one mapping. Myronenko and Song [13] introduce a probabilistic method, called the Coherent Point Drift (CPD) algorithm, for both rigid and non-rigid point set registration. The CPD consider the alignment of two point sets as a probability density estimation problem. These methods have very low dependence with image information and do not need a complex description of the feature points; they turn the process of points matching to be an iteration of the objective function optimization. The objective function is usually a function about image transformation matrix with lower errors or time expense, such as [14] [15].

Daubechies introduced a typical choice for wavelet function in her well-known textbook [16]. This function is represented by the second derivative of Gaussian, and sometimes called the Mexican hat function because it resembles a cross section of a Mexican hat. We find that the Mexican hat function is a function that can get good performance in both area detection and point detection, because of the relationship between the Mexican hat function and the difference of Gaussian (DoG). For example, the well-known LoG operator which is introduced by Marr and Hildreth as an edge detector [17] produces a circularly symmetric Mexican hat.The Mexican hat wavelet is used for feature detection by Yasein [18].

### C. Motivation and Contribution

The distinct objects play a pivotal role in the image registration. A distinct object usually contains obvious features that can be used for image registration, including the edge of the object and the key points on the special positions (the corners, the endpoints and so on). In the area of a distinct object, there would be a set of usable image features, and the positional correspondence is maintained when the image changes happen. The local areas usually have a clear physical meaning which means that they usually represent some distinct objects that contain dense key points. A matching of a local area pair implies a matching of a point group pair. We use the method that combines the two classes of features for a precise registration. We find the distinguishing feature through local area detection. In the local area, a group of key points is used for the description and matching of distinct objects. The point group is much more reliable than the border of the local area because the feature point descriptor contains more invariable properties.

In addition, it is well-known that the computational complexity of many algorithms is the higher-order function of problem size or data scale. Thus, if a large problem is decomposed into the multiple small problems, the whole computational time for solving it will be reduced. More concretely speaking, in the registration of two (multiple) images, the large number of feature points or the high complexity of feature describer seriously affects the computational volume of a matching algorithm. Thus, our objective is to find such an effective strategy for decomposing feature points into several sets that the number of matching operations or the computational volume of a matching algorithm is significantly decreased in the cost of the slight loss of performance. Interestingly, the proposed grouped matching method achieves this objective by grouping the feature points into the local areas. In order to keep the performance of the proposed algorithm, a good transformation matrix is chosen by a strategy named grouped sample consensus (GCS).

**Figure 1:**
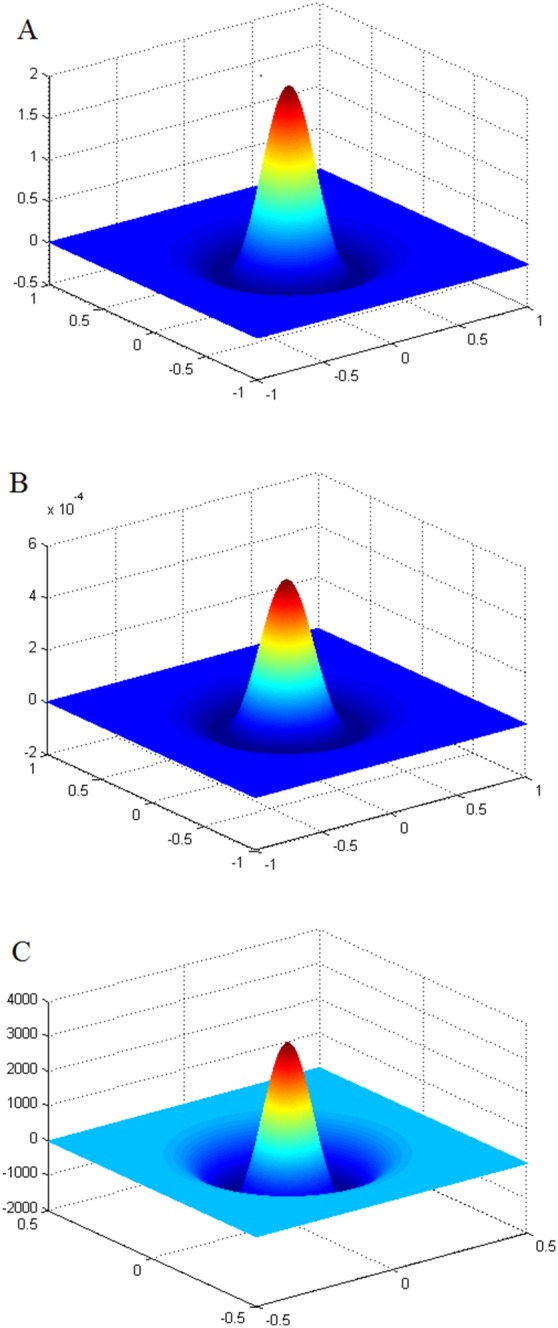
Wavelets of the Mex, LoG and DoG operator.

There are two contributions in our work. The first major contribution is to give a common simplification operator, which is called Mex operator. Figure 1 shows three diagrams for the Mex, LoG and DoG operator (Figure 1 A–C). It can be seen that the Mex operator is well approximate to the LoG and DoG operator, since these three operators produce a circularly symmetric Mexican hat. Especially, when we require a method that combines two or more classes of features, it is necessary to use a common operator similar to the LOG. Interestingly, the DoG operator is generally used for extracting point features, while our operator can extract both area and point features. We give two methods that using the Mex operator for detecting the features and the methods achieve good performance.

The second contribution is that we propose a fast and robust method for estimating the transformation matrix. An image transformation matrix is estimated by a group of points and then the best one is chosen through competition of a set of the transformation matrices. The transformation matrix is well selected by the Grouped Sample Consensus (GCS), which estimates the global transformation matrix through the local sample-data. Importantly, the GCS can also remove the outlier features.

The rest of this paper is organized as follows: Section II presents the Mex operator’s generating and its performance in the feature detection. Section III discusses the grouped feature matching and the transformation matrix estimation through GCS. The Experimental setup and performance evaluation are presented in section IV. Finally, we conclude our work in section V.

## Feature Point Detection

### A. Mexican Hat Operator

In Lowe’s work, we can get the well known DoG operator:
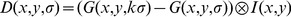
(1)Where, 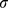 is the space factor, *k* is the factor using for varying scale space. The relationship between the DoG operator and the LoG operator is described by:
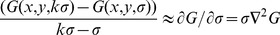
(2)Where 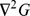 is the LoG operator.



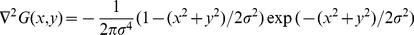
(3)Hence, we have:
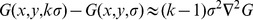
(4)Then we get a slightly simplified function that can replace the DoG:
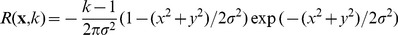
(5)Where 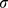 is a scale factor and 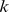 is a variation factor of 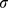 using for varying scale space, here we take 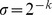. k can be consecutive positive integers bigger than 1. Moreover, 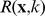 is a Mexican hat function that can be called the Mex operator in this work. It is worth mentioning that 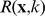 is also a simplification of the operator defined in [10]. The two-dimensional waveform of the Mex operator, LoG operator and DoG operator are shown in figure 1. The Mexican hat function can be seen as the second order derivative of Gaussian function, and its local extreme points can be seen as the extreme points in DoG scale space. So the Mex operator can simplify the operation in the scale space feature detection.

### B. Local Area Detection

**Figure 2:**
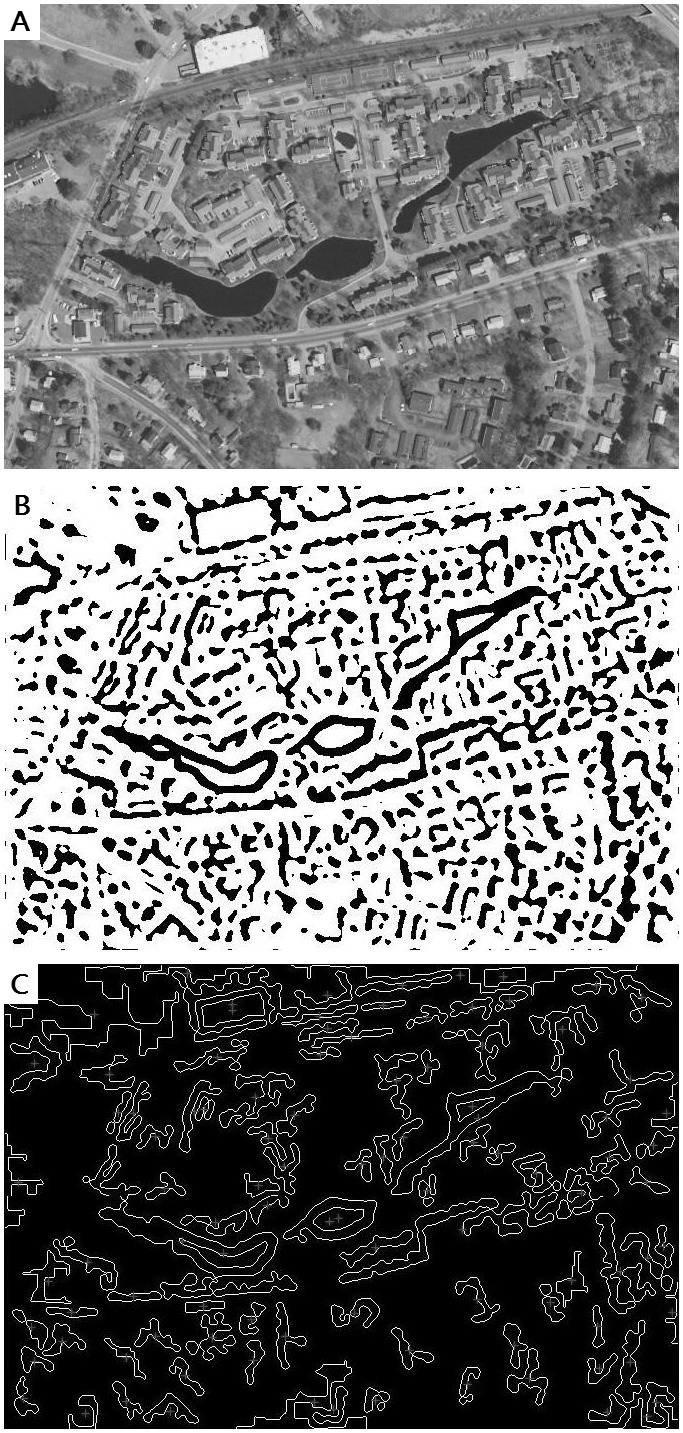
Procedures of the local area detection.

Zero-crossing is a common method for edge detection. The crossing of the second derivative curve and number axis reflect the dramatic intensity change in the image. As a result, pixel points with distinctive features could be detected on this basis. The function of Zero-crossing method is shown as:
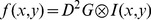
(6)Where 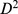 is the second derivative operation, 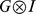 is the smoothing of the image with Gaussian filtering. Owing to the fact that the maximum gradient can only be achieved in the vertical direction of the intensity change, it is not sufficient to choose zero-crossings of the second derivative in any direction. The second derivative is non-zero in the direction that are perpendicular to the direction of the intensity change. In order to solve this problem, 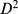 is replaced by operators that do not rely on the direction, such as the LoG operator in Marr’s work. In our work, we take Mex operator, whose distribution remains rotation invariance no matter in space domain or in the frequency domain. Figure 2 shows the procedures of the local area detection on an input image (Figure 2 A).

1. Image filtering is conducted using the Mex operator; thresholding is taken on the image grayscale (Figure 2 B). Since zero is represented by an intermediate gray after the filtering, the very positive values appear white, and the negative ones appear black. We give polar values to describe the white and black pixel in order to make the boundaries distinct.
2. Zero-crossing detection is conducted, and the closed contours are formed by connecting the zero crossing points (Figure 2 C). Zero-crossing patterns, which are composed of signs of pixel values of the filtered image, are detected along both vertical and horizontal directions. The pixel that we mark as an edge point satisfies the following two conditions: the pixel is a zero-crossing point (significant change of the convolved image); the pixel is the closest one to the virtual zero plane of the convolved image among its eight neighbors. When the detecting goes to an endpoint of the edge, a low-threshold satisfied point is chosen and the detection starts from the new point until a closed area is achieved. The low-threshold for edge strength (here we use the Mex value of the pixel) is set to 0 in our work.
3. If an area we get does not encompass sufficient feature points, this area is removable. For example, we eliminate the areas that contain less than 3 points, because the transformation matrix estimation needs at least 3 pairs of matching points. The bigger value the threshold is set as, the less number of the regions we get.

### C. Feature Point Detection

**Figure 3:**
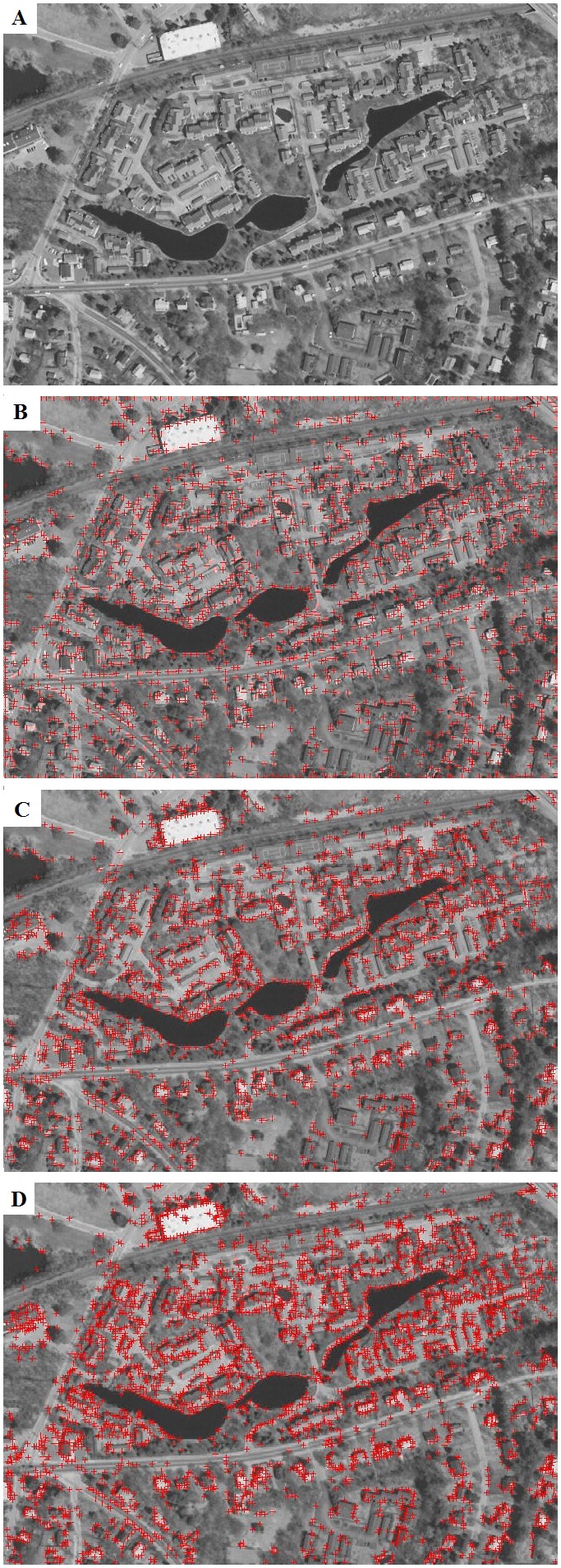
Feature point detection in different scale spaces.

Lecture [19] has proposed a general model which is based on the observation that the curvature response of the feature detectors roots in the difference of two low-pass responses of different bandwidths. The response of the feature detector, denoted by 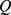 at location 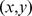 is defined as:
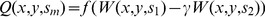
(7)Where 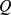 is the representation of the descriptions and the positioning by employing the difference of the filter response in the different scale space. 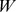 is the normalization of the low-pass filtering in discrete scale space, like 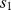 and 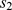, *f* is the modulation function, and 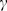 is a constant. Using Mex operator in (6), we get the feature point detection method:
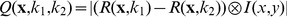
(8)

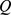
 is taken for calculating the difference between two neighboring spaces.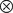 refers to the convolution of image 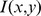 with the Mex operator. We only concern about the extremum in a local area and do not care about whether the extreme value is positive or negative. So we take 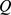 as the absolute value of the difference between the filter response in scale 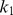 and 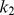. 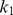 and 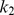 represent two neighboring space, and they are not specific scales that have to be determined by the user. Figure 3 shows the result of the local extrema detection on an input image (Figure 3 A) in scale space with 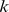 = 2, 3 and 4 (Figure 3 B–D).

## Grouped Points Matching

### A. Area Matching

The majority of the feature matching consists of three steps: local area matching, feature points grouping and points matching. In the step 1, the geometric centers of the detected areas are seen as the interesting points. The interesting points in the two images are matched through equation (8). The equation is a fast and stable application method based on the voting strategy, which uses the spatial geometrical relations among the interesting points for image feature matching.
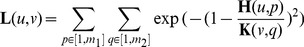
(9)Where, 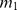 and 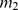 are the number of standby registration points in the master image and the slave image respectively. 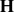 and 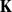 are the matrix formed by the distance of all these points in the images respectively. 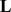 is the voting matrix. If 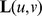 is the maximum in row 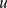 and line 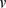 of matrix 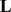, and 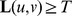, 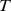 is a constant, then the 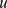 *-*th point in the master image and the 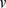-th point in the slave image is a pair of matching points.

### B. Grouped Points Matching

Generally, feature points partitioning or grouping is an effective method to increase the matching efficiency. After the local area and feature points are achieved, image registration would be conducted by combining the two classes of features. First, the matching of the local area is conducted. Then the points are grouped according to the areas, and matched in each group.

**Figure 4:**
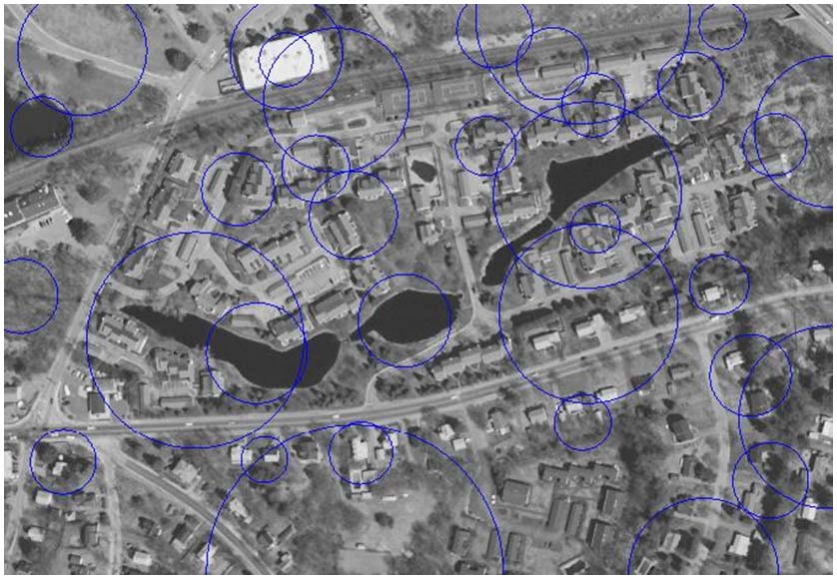
Image partitioning.

In view of the fact that the accuracy of area detection is an effective factor on image partitioning and points grouping, we use a standard circular region that takes place of a detected area to encompass the pixels as shown in Figure 4. A circle’s center is the geometric center of a detected local area; its radius is the average of the distances from all the points on the area contour to the geometric center of the area. It is convenient because we only need to compare the distance from the point to the center with the radius when distinguishing the point in or not in the region.

**Figure 5:**
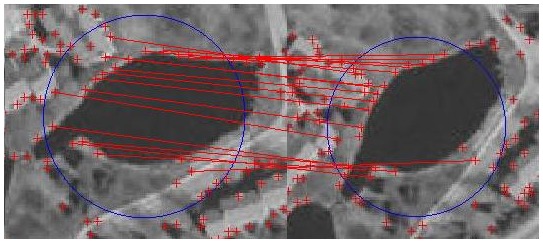
Points matched in a pair of matched regions.

Figure 5 shows the points matched in a pair of matched regions. The image is partitioned into several circular regions and the points are grouped according to regions. The points in the same region are in the same group, and the points that do not in any region are not grouped. The computation speed can get faster since that, the number of the points is small in each group. We append the regional center to the point’s location properties so that the points in one region match with the points in the corresponding region.

The time complexity of the grouped feature point matching is 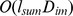, where 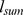 is the number of all the feature points and 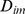 is the dimensionality of the descriptor. So when the feature point descriptor is given and fixed, the time complexity depends on the number of the points. After points grouping, the time complexity is 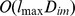, where 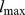 is the biggest number of the points in all the groups. Obviously, 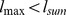. So in this work, the time complexity of the feature point matching depends on the group with the biggest number of points rather than all the feature points.

### C. Transformation Matrix Estimation

After the areas matching and points matching, the transformation matrix would be achieved by optimizing the matching results in different groups, and the procedures are shown as follows.

1. Calculating the transformation matrices and the root mean square errors (RMSE) taken through the matching points in each group respectively.
2. The transformation matrix of a random group is employed to verify all the feature point pairs, and reserves the point pairs meeting the following requirements as the candidate point pairs set 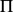 whose size is *sum*:
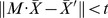
(10)Where 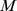 is the transformation matrix of a random point group, 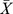 and 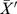 are the points in the master and the slave image, 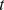 is the fault-tolerant error. If 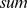 is bigger than the threshold, let 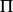 be a candidate inner set, then return to 1); otherwise, return to 2).
3. After λ iterations, when the *sum* remains unchanged, takes the matrix with the biggest size of 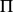 and receivable RMSE as the transformation matrix of image matching.

Since the strategy is an application of the Random Sample Consensus (RANSAC) in the grouped points matching we call it Grouped Sample Consensus (GCS). The matrix in a random group which can satisfy the requirement (9), which has low RMSE and sufficient feature points, can be seen as a candidate matrix. The final transformation matrix is the one with the least RMSE and the largest number of matching points in all the candidate matrices. That is, the whole algorithm would not collapse if there is one matrix fulfill the conditions.

The time complexity of GCS is 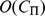, 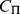 is the probability in procedure 2). Interestingly, GCS does not just remove outlier points, it is also a solution on regions mismatching. When there is a mismatching of a region pair, the candidate matrix based on that pair can’t provide inner point pairs and the candidate matrix is rejected.

## Experimental Results

### A. Data Set

**Figure 6:**
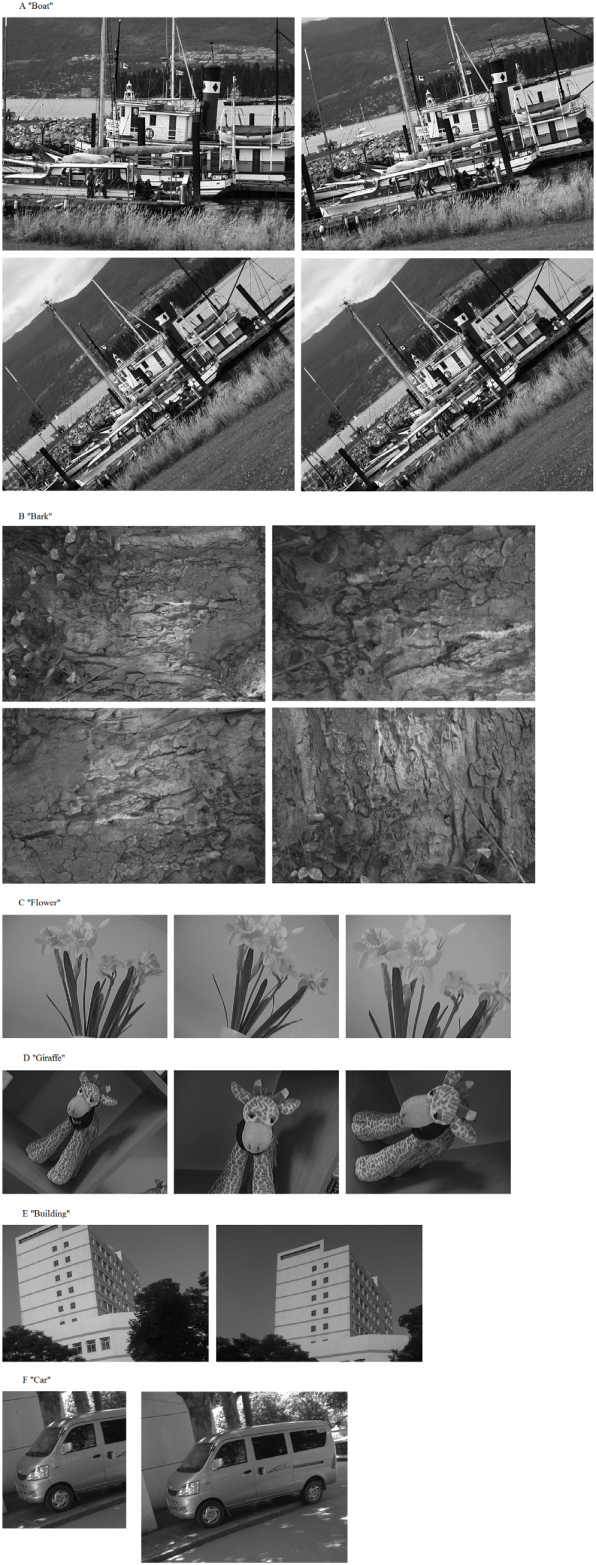
Text images.

We use 12 pairs of images for the matching work shown in Figure 6. Some are from a Mikolajczyk’s testing image set which are frequently used in image processing (Figure 6 A shows the image “Boat” and its three matching images. Similarly, the Figure 6 B denotes the image “Bark”). Some are optical pictures (Figure 6 C–F). All the images are taken due to the real changes through different focal distance and perspectives. So they contain the images with a great majority of rigid changes and some slightly perspective transformations.

### B. Performance

**Figure 7:**
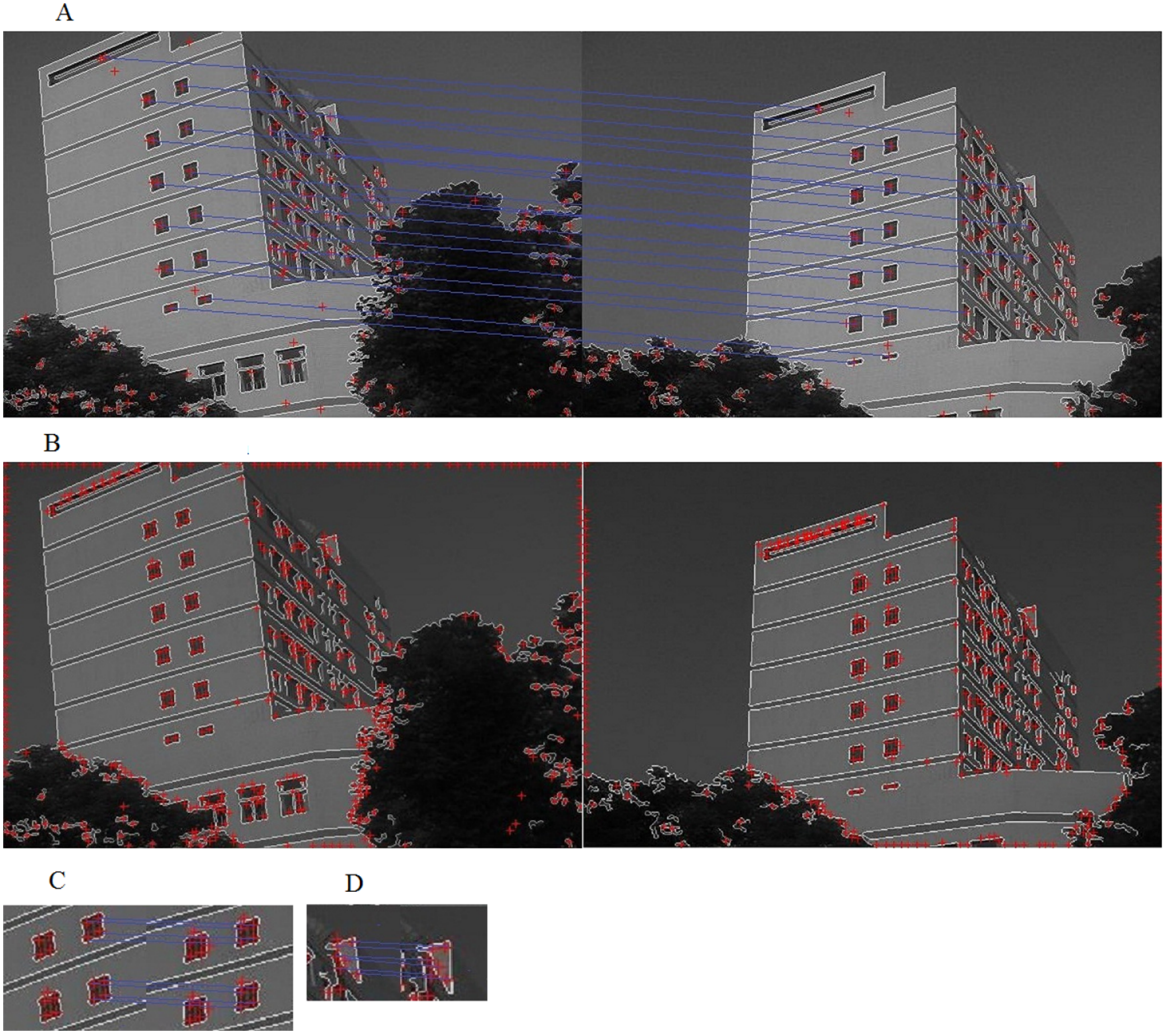
Performance of the image “Building” registration.

Figure 7 shows the result of the test image “Building” (Figure 6 E) registration, the “Building” contains optical images from different viewing angles.

The master image and the slave image are both partitioned into 20 regions after local area detection and matching (Figure 7 A). Feature points are detected (Figure 7 B) and point matching is conducted in each region (Figure 7 C and D). Each region can get a candidate transformation matrix and the optimal matrix is achieved by GCS. Comparison on the registration accuracy and efficiency among our work, area-based LoG operator, and point-based SIFT operator has been carried out for Table 1. The repetition is the ratio of matched points in the whole points.

**Table 1:** Registration result.

Operator	Number	Repetition	RMSE	Time
Mex	1078	72%	0.0726	120s
SIFT	1204	48%	0.0634	513s
LoG	73	/	0.7430	13s

The local area obtained by the LoG operator is so small in number with precise positioning that there is little sense in comparing repetition rate, and it is difficult to achieve good results in registration accuracy. The Mex operator can provide more image features and higher accuracy than the LoG operator. When compared to the SIFT operator, the grouped matching strategy achieves a higher repetition rate and runs much faster. All the experiments in our work were executed on an Intel Pentium(R) Dual-Core CPU 2.5 GHz computer with 2 G RAM in a Matlab environment.

### C. Analysis of Area Detection

Since the local area detection and matching are crucial for the registration method, we discuss their influences as follows. In testing image pair “car” that contains scale change and clipping, we set different values for the threshold in local area detection to get different number of features.

For simplicity, we use the number of the pixels encompassed in the area for the threshold. Four different thresholds are chosen in the master image and the detection threshold is set to 200 in the slave image. The details of the matching are shown in Table 2.

**Table 2:** Feature matching result.

Threshold	Matched area	Points in area	Matched points	Time
200	17	225	237	34s
400	9	162	246	48s
800	4	120	235	73s
1600	3	98	228	82s

It can be seen that, the number of the detected area has an indirect relation to the feature point matching. However, when the matched areas are small, we still get sufficient inner points because of the GCS strategy that estimates the global transformation matrix through the local sample-data. Although the running time of our algorithm becomes longer because the cost on inner choosing gets higher, the algorithm would be useful even though only one detected area meets the condition under which this region encompass sufficient a number of the good feature points that can be used to estimate a transformation matrix with receivable accuracy.

### D. Comparison

In order to testify the algorithm further, we use four quantitative measures to evaluate its performance: the recall, the precision, the RMSE and the running time. The recall is defined as follows. If 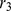 pairs of points are matched, and actually there are 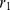 and 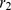 points with matching alternatives in the two images, then the recall is 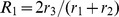. The precision is given by 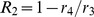, in which 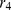 is the number of the wrong matched pairs. The higher the recall and the precision behave, the more stable and accurate the matching methods are.

Comparisons between our work and the most popular point detection and matching methods are taken. The alternatives are the SIFT, and the scale invariant Harris. These methods use RANSAC for transformation matrix estimation, while our work is exploiting the Mex operator with GCS. In our experiments, the factor *k* is taken as 2, 3 and 4, in order to construct a range of scale spaces. We determine the factor *k* based on only one fact that *k* should be consecutive positive integers bigger than 1. We chose the range 2, 3 and 4 because that the Mex operator is mostly similar to the LoG when *k* = 2. Since the Mex operator can approximate the second order derivative of Gaussian function, we actually detect key points in 4 levels of different Gaussian space like SIFT. And the other operators in our experiments use the same range scales for fairness and universality. Finally, all the three methods take the SIFT descriptor for the feature point description.

**Figure 8:**
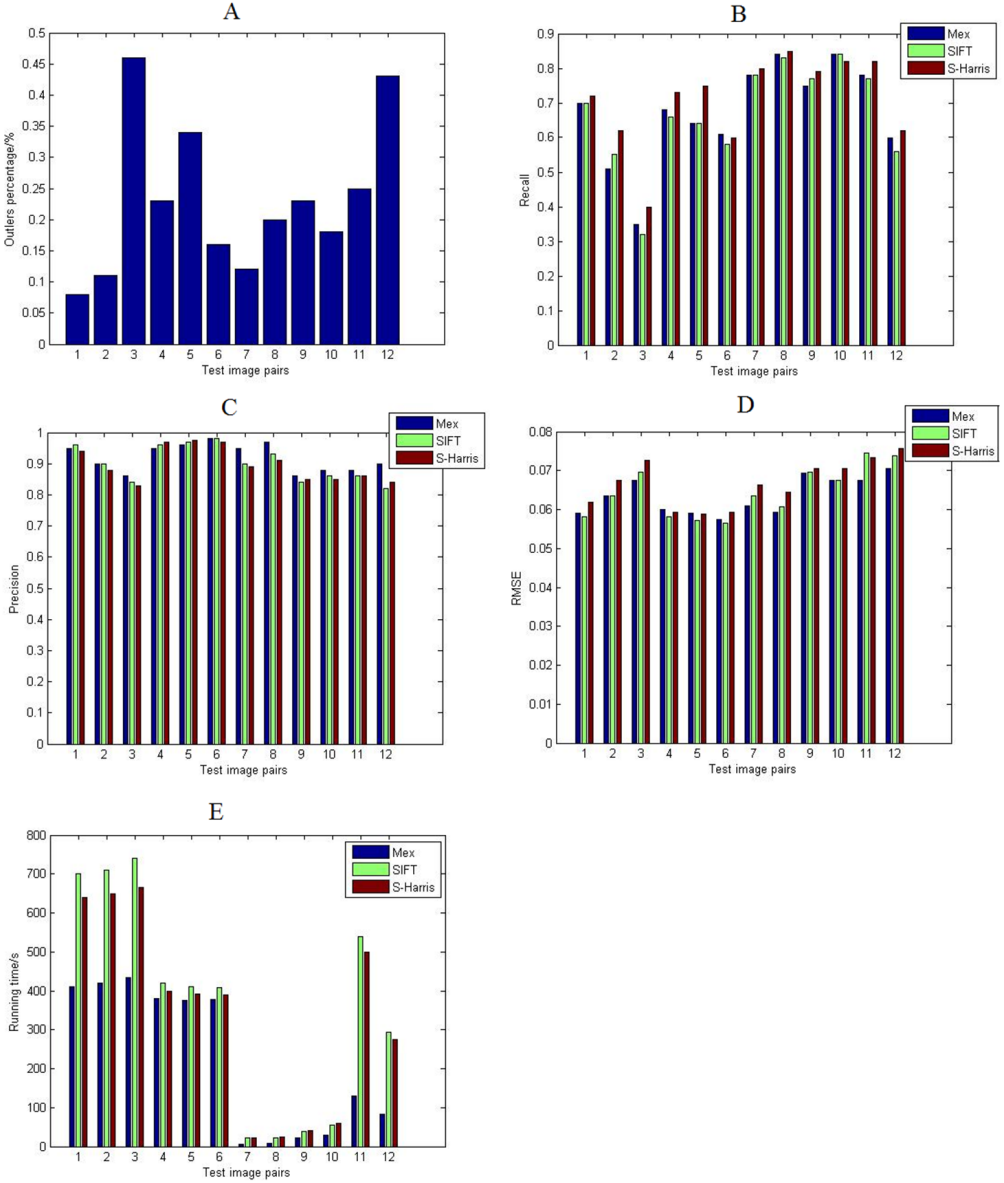
Comparison results.

The results are shown in Figure 8.The numbers on the x-axes in Figure 8 are corresponding to the 12 pairs of images in Figure 6. For example, the 1, 2 and 3 are three matching results for the “Boat”.

It can be seen that, the proportion of the outliers becomes higher when the scale transformation happens (Figure 8 A). The threshold in GCS depends on the robustness required in the application, and we set it to 50% of all detected points, which can sufficiently satisfy the image registrations in our experiments.

The S-Harris, short for the scale invariant Harris operator, achieves the best performance on recall (Figure 8 B). The Mex operator in our work and the SIFT get the similar result. That is because, the Mex operator and the SIFT detect the points in the scale space pyramid, and the points are a union of several space. The recall is not connected with the proportion of the outliers, but is influenced by the number of the feature points. The third pair has the lowest recall because the matching images in that pair have the most feature points. The matching images in “flower” also contain the scale changes, but the recall of the Mex is higher than the S-Harris since the images take the simple textures. It can be seen that, the Mex operator in our work achieves the same recall as the other scale space based-methods. Moreover, the Mex operator’s recall is higher than the SIFT’s in the matching of rotation changes.

Our method achieves the best performance on precision for some testing pairs (Figure 8 C), for example, the matching images “giraffe”. There are salient objects in the “giraffe” so that the features, both the local areas and the points, are detected and worked effectively. The GCS gets the best result when the two classes of features are combined; there is about 10% more on precision than that in the other methods. In some testing pairs, the “bark” for example, the textures in the images are discrete, so the GCS strategy is not efficient and regresses to the normal RANSAC as in the other methods.

The RMSE is directly connected with the precision and the detecting accuracy. The RMSE (Figure 8 D) shows that, the profile is corresponding to the precision curve (Figure 8 C). It is an evidence presents that the Mex operator achieves as accurate detecting result as the SIFT gets.

Another advantage of our method is on running time (Figure 8 E). Like the precision, the GCS strategy performs much better when the two classes of features are working together. The points are grouped and the running time decreases according to 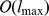.

Table 3 shows the average results of the comparisons. It can be seen that, our algorithm gets better performance than the famous SIFT and S-Harris on the registration accuracy, and achieves much better results in running time.

**Table 3:** Average result.

Operator	Recall	Precision	RMSE	Time (s/point)
Mex	67%	92%	0.0642	0.1391
SIFT	66%	90%	0.0644	0.5371
S-Harris	71%	89%	0.0662	0.4686

## Conclusion

In this work, we provide a Mexican hat function-based operator on image feature detection. We use the operator for achieving an improved zero-crossing method of local area detection and detection of feature point is conducted in different scale space. By combining the two classes of image features, we propose a grouped feature point matching strategy and a grouped sample consensus strategy to achieve a fast and accurate image registration.

The future work for the research is that, the local area changes in the affine transformation so the regions we take to group the points can’t be circles. An affine invariant region should be an ellipse, the orientation and the accuracy of edge detection is crucial to the feature matching.
